# Classification of Primary Cerebral Lymphoma and Glioblastoma Featuring Dynamic Susceptibility Contrast and Apparent Diffusion Coefficient

**DOI:** 10.3390/brainsci10110886

**Published:** 2020-11-20

**Authors:** Felix Eisenhut, Manuel A. Schmidt, Florian Putz, Sebastian Lettmaier, Kilian Fröhlich, Soheil Arinrad, Roland Coras, Hannes Luecking, Stefan Lang, Rainer Fietkau, Arnd Doerfler

**Affiliations:** 1 Department of Neuroradiology, University of Erlangen-Nuremberg, Schwabachanlage 6, 91054 Erlangen, Germany; manuel.schmidt@uk-erlangen.de (M.A.S.); hannes.luecking@uk-erlangen.de (H.L.); stefan.lang@uk-erlangen.de (S.L.); arnd.doerfler@uk-erlangen.de (A.D.); 2 Department of Radiation Oncology, University of Erlangen-Nuremberg, Universitaetsstrasse 27, 91054 Erlangen, Germany; florian.putz@uk-erlangen.de (F.P.); sebastian.lettmaier@uk-erlangen.de (S.L.); rainer.fietkau@uk-erlangen.de (R.F.); 3 Department of Neurology, University of Erlangen-Nuremberg, Schwabachanlage 6, 91054 Erlangen, Germany; kilian.froehlich@uk-erlangen.de; 4 Department of Neurosurgery, University of Erlangen-Nuremberg, Schwabachanlage 6, 91054 Erlangen, Germany; soheil.arinrad@uk-erlangen.de; 5 Department of Neuropathology, University of Erlangen-Nuremberg, Schwabachanlage 6, 91054 Erlangen, Germany; roland.coras@uk-erlangen.de

**Keywords:** primary central nervous system lymphoma, glioblastoma, multimodal, MRI, ADC, DSC

## Abstract

This study aimed to differentiate primary central nervous system lymphoma (PCNSL) and glioblastoma (GBM) via multimodal MRI featuring radiomic analysis. MRI data sets of patients with histological proven PCNSL and GBM were analyzed retrospectively. Diffusion-weighted imaging (DWI) and dynamic susceptibility contrast (DSC) perfusion imaging were evaluated to differentiate contrast enhancing intracerebral lesions. Selective (contrast enhanced tumor area with the highest mean cerebral blood volume (CBV) value) and unselective (contouring whole contrast enhanced lesion) Apparent diffusion coefficient (ADC) measurement was performed. By multivariate logistic regression, a multiparametric model was compiled and tested for its diagnostic strength. A total of 74 patients were included in our study. Selective and unselective mean and maximum ADC values, mean and maximum CBV and ratio_CBV_ as quotient of tumor CBV and CBV in contralateral healthy white matter were significantly larger in patients with GBM than PCNSL; minimum CBV was significantly lower in GBM than in PCNSL. The highest AUC for discrimination of PCNSL and GBM was obtained for selective mean and maximum ADC, mean and maximum CBV and ratio_CBV_. By integrating these five in a multiparametric model 100% of the patients were classified correctly. The combination of perfusion imaging (CBV) and tumor hot-spot selective ADC measurement yields reliable radiological discrimination of PCNSL from GBM with highest accuracy and is readily available in clinical routine.

## 1. Introduction

Glioblastoma (GBM) is the most common malignant central nervous system neoplasm [1]. Despite current advances in multimodal treatment—including radical surgical resection, radiation and chemotherapy—this glioma subtype remains highly aggressive with a poor prognosis and a high reoccurrence rate [2,3]. Primary central nervous system lymphomas (PCNSL)—up to 95% are diffuse large B-cell lymphomas—are also aggressive tumors with a median untreated survival of just a few months. Treatment options, e.g., corticosteroids, methotrexate-based chemotherapy and radiation, are effective attempts, but the relapse rate is high [4]. Because of their typical radiological features PCNSL (basal ganglia or periventricular white matter location, homogenous contrast enhancement, rarely hemorrhage or necrosis) and GBM (peripheral ring enhancement, intralesional hemorrhage, central necrosis) can usually be differentiated.

However, untypical imaging appearance of PCNSL is frequent and can complicate diagnosis. Therefore, and because patient management as well as treatment options differ significantly between GBM [5] and PCNSL [6], a reliable radiological classification is of great clinical relevance. Consequently, several approaches aim for precise differentiation via multimodal magnetic resonance imaging (MRI): diffusion-weighted imaging (DWI) [7], dynamic susceptibility contrast (DSC) perfusion imaging [8], susceptibility-weighted imaging (SWI) [9] and multiparametric models combining those sequences [10,11] demonstrated promising results regarding the correct radiological classification of GBM and PCNSL. In addition, three meta-analyses recently confirmed multimodal MRI as a reliable assessment tool for GBM and PCNSL discrimination [12,13,14].

Even so, there is a wide variety of measuring methods for DWI and DSC imaging—e.g., approaches placing multiple intralesional region of interests (ROI) and calculating means, contouring the whole contrast enhancing lesion or placing selective ROIs have been suggested. Furthermore, multiple distinct variables, means, ratios and scoring systems have been applied and evaluated to predict GBM and PCNSL.

In this paper, we want to present our approach for a fast and convenient differentiation of glioblastoma and primary central nervous system lymphoma by using selective DWI ROI measurements in combination with DSC perfusion data that is applicable in the clinical routine. In addition, we compare our approach to unselective, whole-lesion measuring and evaluate its predictive accuracy regarding tumor neuropathology.

## 2. Materials and Methods

### 2.1. Patients

Patients with histologically diagnosed primary central nervous system lymphoma from 2014 to 2019 were considered. Age and gender matched glioblastoma patients were selected from 2014 to 2019.

All patients included underwent multimodal 1.5 or 3 T MRI ahead of any medical treatment.

Informed consent was obtained from all patients.

### 2.2. Acquisition and Postprocessing

#### 2.2.1. MRI

The 1.5 T, as well as 3 T MRI, was performed at the department of neuroradiology of our hospital. The 1.5 T MRI was performed on a Magnetom Aera; 3 T MRI was performed on a Magnetom TrioTim (both Siemens Healthineers AG, Erlangen, Germany).

#### 2.2.2. Imaging Protocol and Sequence Details

All performed MRI examinations included: a fluid-attenuated inversion recovery (FLAIR) sequence, a native T1 weighted sequence, a T2 turbo spin echo sequence, a T2-star weighted/gradient-echo imaging sequence (sensitive for hemorrhage and hemosiderin deposits), a DWI sequence, DSC perfusion imaging with leakage correction and a isotropic contrast-enhanced magnetization prepared-rapid gradient echo (MP-RAGE) T1 sequence. For further details see Table 1.

At the fourth time point of DSC perfusion imaging, weight adapted 0.5 mmol/mL DOTAREM (Guerbet, Villepinte, France) or DOTAGRAF (Jenapharm GmbH & Co. KG, Jena, Germany) was intravenously administrated at a flowrate of 3.5 mL/s, followed by a 20 mL saline flush.

#### 2.2.3. Postprocessing

Datasets were preprocessed in a standardized manner using the predefined procedure of the MR neurology workflow of commercially available post-processing software (syngo.via, Siemens Healthineers AG, Erlangen, Germany). Background segmentation was performed to remove extracranial tissue using an automatically detected noise threshold. To maintain data integrity and limit confounding factors, the automatic presets for spatial and temporal smoothing were applied. The prebolus range was adjusted when necessary. The raw signal was converted “SI to delR2”, i.e., into relative change in R2-star (reciprocal of T2-star) versus time. Automatic arterial pixel selection was chosen for computing a local arterial input function (AIF). The AIF was generated automatically by the software for each individual dataset using a global clustering method which examines the time series for all voxels and identifies a suitable AIF. Correction for T1 leakage effects was ensured by using the provided algorithm of syngo.via. Then, DSC CBV perfusion and ADC maps were automatically co-registered with T1 contrast enhanced MP-RAGE images using commercially available post-processing software (syngo.via, Siemens Healthineers AG, Erlangen, Germany) and the implemented linear registration tool.

Three ROI measurements were performed on each data set:

The first ROI (ROI selective) was placed carefully in the contrast enhanced lesion area with the highest CBV value avoiding vessels, cystic or necrotic areas, cerebrospinal fluid (CSF) and bone.

The second ROI (ROI unselective) comprised the whole contrast enhancing lesion including necrotic or cystic areas without regard to CBV values.

**Figure 1:**
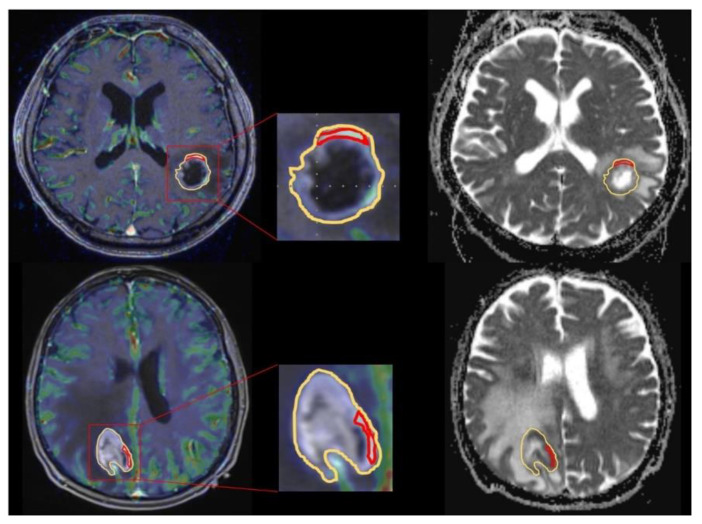
Exemplary apparent diffusion coefficient (ADC) and cerebral blood volume (CBV) measurement in a glioblastoma (GBM) and primary central nervous system lymphoma (PCNSL) patient. Exemplary ADC measurement of a selective region of interest (ROI)(red) in the contrast enhanced tumor area with the highest CBV and a unselective ROI (yellow) comprising the whole contrast enhancing lesion for a GBM patient (upper line) in comparison to a PCNSL patient (bottom line). After placing the ROIs carefully in the fused CBV × contrast enhanced T1 sequence map (right) ROIs are copied to the exact same position in the corresponding ADC maps (left).

Afterwards ROIs were copied to the exact same region in the corresponding ADC map. Figure 1 shows exemplary ROI measurements in a GBM patient as well as a PCNSL patient.

A third ROI was placed in the co-registered DSC CBV perfusion maps in the contralateral hemisphere—mirrored along the midline (falx cerebri)—in unaffected white matter with the possibility of manual adjustments to avoid vessels, CSF and bone. Image postprocessing and ROI placement took approximately 4 min.

### 2.3. Data Evaluation

All data sets were retrospectively analyzed with commercially available, clinical software (syngo.via, Siemens Healthineers AG, Erlangen, Germany).

Quantitative assessment:

Minimum, maximum and mean values were measured of selective and unselective ADC ROIs. Minimum, maximum and mean values were measured for all CBV ROIs. Selective ADC values were measured in the selected, contrast enhancing tumor areas with the highest CBV values. Unselective ADC and all CBV values were measured in the whole contrast enhancing lesion area without regard to CBV values, including necrotic or cystic tumor areas. Mean CBV values were measured in unaffected, healthy white matter of the contralateral hemisphere avoiding vessels and CSF.

### 2.4. Statistical Analysis

Minimum, maximum and mean selective and unselective ADC and minimum, maximum and mean CBV values were analyzed by use of descriptive statistics and tested for normal distribution by using the D’Agostino–Pearson test (if *p* > 0.05, normality was accepted).

Selective minimum, maximum and mean ADC values as well as unselective minimum, maximum and mean ADC values were compared between patients with PCNSL and GBM by use of an unpaired, two-tailed *t*-test.

Minimum, maximum and mean CBV values were compared between patients with PCNSL and GBM by use of the Mann–Whitney U test. Mean CBV values in the contrast enhancing lesion to mean CBV values in contralateral healthy white matter ratios (ratio_CBV_) were computed for both PCNSL and GBM and tested by use of the Mann–Whitney U test.

Receiver operating characteristics (ROC) analysis was performed for selective and unselective minimum, maximum and mean ADC values as well as CBV values.

For multiparametric analysis a multivariate logistic regression analysis was performed.

Statistical analysis was performed with GraphPad Prism 8 (GraphPad Software, San Diego, CA, USA) and Excel (Microsoft, Redmond, WA, USA). Multivariate logistic regression analysis was performed with SPSS 19 (IBM, Armonk, NY, USA).

*p* values less than 0.05 were considered statistically significant. *p* values less than 0.05 are marked with “*”, less than 0.01 with “**”, less than 0.001 with “***” and less than 0.0001 with “****”.

## 3. Results

### 3.1. Patients

In total, 74 patients (49 males, 25 females, median age 68.3 years) with full multimodal MRI ahead of any medical treatment were included in our study: 37 patients with histologically diagnosed PCNSL (24 males, 13 females, median age 68.7) and 37 age and gender matched patients with histologically diagnosed GBM (25 males, 12 females, median age 67.9).

### 3.2. DWI Results

**Figure 2:**
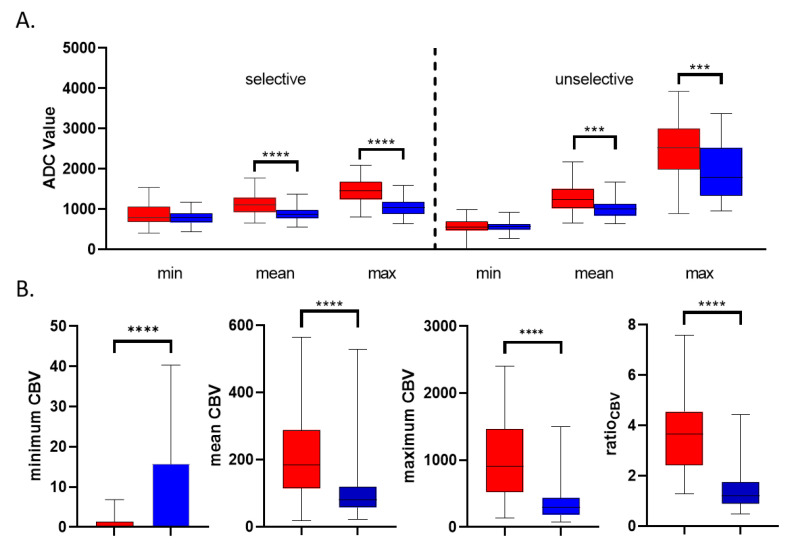
Box plot analysis of ADC and CBV measurement in GBM and PCNSL patients. (**A**) Box plot of apparent diffusion coefficient analysis of selective and unselective minimum, mean and maximum ROI measurement in patients with GBM (red) and PCNSL (blue). (**B**) Box plot of DSC CBV imaging analysis in patients with GBM (red) and PCNSL (blue). *p* values less than 0.05 were considered statistically significant. *** *p* < 0.001, **** *p* < 0.0001.

**Figure 3:**
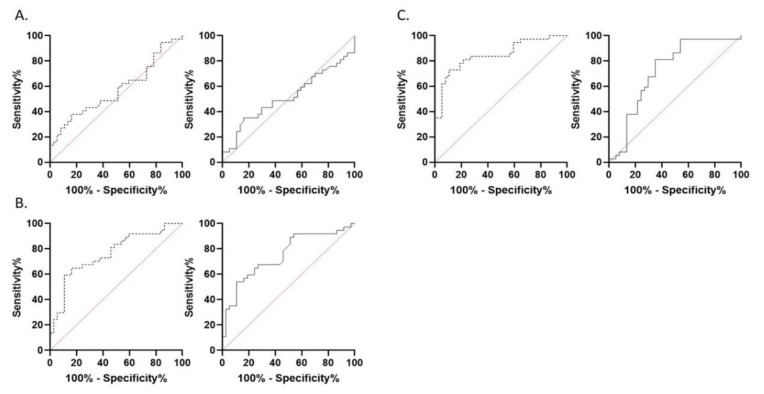
Receiver-operating-characteristics (ROC) analysis of ADC measurements for differentiation of GBM and PCNSL. ROC analysis of apparent diffusion coefficient of selective (dotted line) and unselective (solid line) ROI measurement for differentiation of patients with GBM and PCNSL by minimum ADC (**A**), mean ADC (**B**) and maximum ADC (**C**) values.

Selective minimum, maximum and mean ROI measurements showed a Gaussian distribution (*p*_sel min PCNSL_ = 0.91; *p*_sel min GBM_ = 0.23; *p*_sel max PCNSL_ = 0.38; *p*_sel max GBM_ = 0.70; *p*_sel mean PCNSL_ = 0.36; *p*_sel mean GBM_ = 0.61). Unselective minimum, maximum and mean ROI measurements showed a Gaussian distribution (*p*_unsel min PCNSL_ = 0.21; *p*_unsel min GBM_ = 0.13; *p*_unsel max PCNSL_ = 0.12; *p*_unsel max GBM_ = 0.85; *p*_unsel mean PCNSL_ = 0.053; *p*_unsel mean GBM_ = 0.32). ROI values for minimum ADC values showed no significant difference in patients with GBM and PCNSL whether selective or unselective ROI measurement was performed (ADC_sel min PCNSL_ = 780.1 ± 175; ADC_sel min GBM_ = 863 ± 252; ADC_unsel min PCNSL_ = 566 ± 137; ADC_unsel min GBM_ = 543 ± 248, *p*_sel ADC min_ = 0.11; *p*_unsel ADC min_ = 0.62). ROI values for maximum and mean ADC values showed a significant difference in patients with GBM and PCNSL in both selective and unselective ROI measurement (ADC_sel max PCNSL_ = 1045 ± 224; ADC_sel max GBM_ = 1445 ± 312; ADC_unsel max PCNSL_ = 1929 ± 760; ADC_unsel max GBM_ = 2503 ± 630, *p*_sel ADC max_ < 0.0001; *p*_unsel ADC max_ < 0.0001; ADC_sel mean PCNSL_ = 893 ± 191; ADC_sel mean GBM_ = 1111 ± 239; ADC_unsel mean PCNSL_ = 997 ± 223; ADC_unsel mean GBM_ = 1272 ± 339, *p*_sel ADC mean_ < 0.0001; *p*_unsel ADC mean_ = 0.0001; Figure 2A). The highest area under the curve (AUC) value was obtained for ADC_sel max_ (0.847) and ADC_sel mean_ (0.762), see also Figure 3. The optimal cut-off value to differentiate GBM from PCNSL was determined (ADC_sel max_ = 1314; ADC_sel mean_ = 1066). This corresponded to the histological diagnosis in 89% of PCNSL patients (33 of 37) and 70% of GBM patients (26 of 37) for ADC_sel max_ and in 81% of PCNSL patients (30 of 37) and 62% of GBM patients (23 of 37) for ADC_sel mean_.

### 3.3. CBV Results

**Figure 4:**
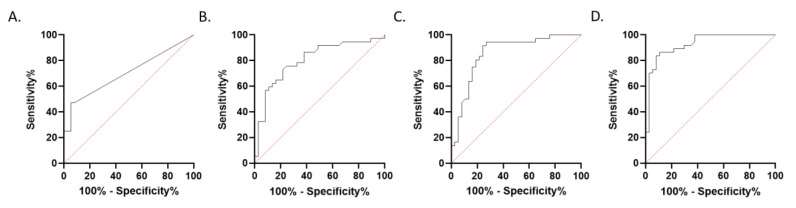
ROC analysis of CBV measurements and the multiparametric model for differentiation of GBM and PCNSL. ROC analysis of DSC CBV imaging for differentiation of patients with GBM and PCNSL by minimum (**A**), mean (**B**) and maximum CBV (**C**) and ratio_CBV_ (**D**).

Minimum and mean CBV measurements of the contrast enhancing lesions and maximum CBV measurements of PCNSL showed no Gaussian distribution (*p*_CBV min PCNSL_ = 0.0001; *p*_CBV min GBM_ < 0.0001; *p*_CBV mean PCNSL_ < 0.0001; *p*_CBV mean GBM_ = 0.009; *p*_CBV max PCNSL_ < 0.0001), mean CBV measurements of the contralateral healthy white matter and maximum CBV measurements of GBM showed a Gaussian distribution (*p*_CBV contra PCNSL_ = 0.058; *p*_CBV contra GBM_ = 0.16; *p*_CBV max GBM_ = 0.25). Minimum, maximum and mean CBV values showed a significant difference in GBM patients compared to PCNSL patients (CBV_min GBM_ = 1.32 ± 5.5, CBV_min PCNSL_ = 15.7 ± 24.6, *p*_CBV min_ < 0.0001; CBV_max GBM_ = 1013 ± 622, CBV_max PCNSL_ = 353 ± 289, *p*_CBV max_ < 0.0001; CBV_mean GBM_ = 211.5 ± 133; CBV_mean PCNSL_ = 104 ± 87; *p*_CBV mean_ < 0.0001). CBV ratio showed a significant difference in GBM patients compared to PCNSL patients (ratio_GBM_ = 3.76 ± 1.6; ratio_PCNSL_ = 1.39 ± 0.8; *p*_ratio_ < 0.001; Figure 2B). The AUC value for minimum CBV measurements was 0.703, for maximum CBV 0.857, for mean CBV 0.804, for CBVratio 0.930; see also Figure 4A–D. The optimal cut-off values to differentiate GBM from PCNSL were determined (CBV_min_ = 4.5; CBV_max_ = 473; CBV_mean_ = 129.5; ratio_CBV_ = 2.2). This corresponded to the histological diagnosis in 43% of PCNSL patients (16 of 37) and 95% of GBM patients (35 of 37) for CBV_min_, in 78% of PCNSL patients (29 of 37) and 78% of GBM patients (29 of 37) for CBV_max_, in 78% of PCNSL patients (29 of 37) and 73% of GBM patients (27 of 37) for CBV_mean_ and in 89% of PCNSL patients (33 of 37) and 84% of GBM patients (31 of 37) for ratio_CBV_.

### 3.4. Multiparametric Assessment

For multiparametric assessment the following five parameters with the highest AUC were chosen: ADC_sel mean_, ADC_sel max_, CBV_mean_, CBV_max_ and ratio_CBV_.

Applying this model allowed the accurate differentiation of GBM and PCNSL in all included patients. The AUC for this five-parameter model was 1.

Table 2 summarizes the results of the PCNSL and GBM classification for both single parameter approaches and the multiparametric model.

## 4. Discussion

In this study, we evaluated two different approaches of ADC ROI measurement—first by unselective contouring the whole contrast enhancing lesion, second by selective ROI placing in the tumor area with the highest CBV value—in combination with DSC CBV perfusion imaging for differentiation of glioblastoma from primary central nervous system lymphoma. Both selective and unselective mean and maximum ADC values showed a significant difference for the two patient groups. In addition, the selective measurement of mean and maximum ADC values in tumor hot-spot areas increased the diagnostic accuracy and minimized measuring deviations compared to the unselective approach. In contrast, the minimum ADC value did not differ between GBM and PCNSL regardless of selective or unselective ROI placing. Furthermore, all CBV parameters—minimum, maximum, mean CBV values in the whole-enhancing lesion and the ratio of mean CBV in the tumor to the mean CBV in contralateral, healthy white matter—showed a significant difference between GBM and PCNSL. Importantly, combination of the parameters with the highest AUC for discrimination of GBM and PCNLS in our single parameter analysis—mean and maximum selective ADC value and mean and maximum CBV and the CBV ratio allowed radiological differentiation of GBM and PCNSL in all included patients.

Recent studies, including three meta-analyses, discuss the value of multimodal MRI for differentiation of PCNSL and GBM. However, there are numerous different approaches and they are only sparsely applicable in the clinical routine: In their meta-analysis Lu et al. evaluated the diagnostic performance of DWI in eight studies with a total of 461 patients [12]. The authors report, that in four of these reports the ROI for ADC measurement was placed in the solid enhancing part of the tumor and in four studies the ROI measured the whole tumor. The reported, measured ADC values were even more heterogenous: in three studies the mean ADC, in two studies the minimum ADC, and in one study each the fifth percentile value of cumulative ADC histogram, the ADC ratio of the enhancing lesion to the contralateral unaffected white matter, and the ADC value of the most strongly enhanced tumor area was used, respectively. In this meta-analysis there was no statistically significant difference between ADC measurements in whole tumor versus in solid portion. In contrast, in our study, the unselective, whole lesion ADC measurement showed higher standard deviations with a consequently lower AUC for lesion differentiation because of the heterogeneity of both PCNSL and especially GBM lesions with their cystic, necrotic and hemorrhagic areas. Therefore, we conclude that selective ADC measurement in the contrast enhanced tumor area with the highest CBV value might be more robust to discriminate GBM and PCNSL. In our analysis selective and unselective ADC values in GBM were higher than in PCNSL. This is in accordance with previous studies reporting lower ADC values in PCNSL due to the increased cellularity compared to GBM [15,16]. In summary, Lu et al. report a moderate diagnostic performance of using a single ADC value for radiologic PCNSL and GBM assessment and recommend the additional use of MR perfusion-weighted imaging to increase the prediction accuracy. This is in accordance with the second meta-analysis, including 14 studies with 598 patients: here, Xu et al. report the highest level of accuracy for distinguishing high-grade gliomas and PCNLS for perfusion weighted imaging [14]. These findings are in line with our study’s result of ratio_CBV_ being the parameter with the highest AUC for lesion discrimination. Thereby, a singular variable enabled correct identification of 89% of our PCNSL and 84% of our GBM patients, respectively. However, especially regarding the substantial difference in patient management and therapy approaches the radiologist should aim for even higher diagnostic accuracy in GBM versus PCNSL discrimination. In 2014, Kickingereder et al. combined mean ADC and mean CBV values with intratumoral susceptibility signals (ITSS) to increase the probability of correctly identifying PCNSL in 95% and GBM in 96% in a total of 47 patients [10]. We agree with the authors that combining multimodal MRI parameters is helpful for precise radiological neuropathological prediction. However, we do not think Kickingereder et al.’s approach is applicable in the clinical routine: on average 11 ROIs (up to 35) were placed in the ADC and CBV maps of each patient to compute means—in contrast, we used only 3 ROIs for ADC and CBV assessment. Thus, our algorithm may easily be introduced into daily clinical routine reports. In 2018, Saini et al. also used multimodal MRI to discriminate GBM and PCNSL [11]: In their cohort of 100 patients, minimum ADC, maximum CBV, back flux exchange rate and ITSS showed a significant decrease in PCNSL compared to GBM. The authors’ multimodal model enabled them to identify 84% patients correctly. In comparison our five-parametric model integrating selective mean and maximum ADC, mean, maximum CBV and the ratio_CBV_ enabled a correct diagnosis in all included patients and thus seems to be more robust for lesion discrimination. In addition—in contrast to Kickingereder’s and Saini’s approach—the GBM and PCNSL patients in our study were correctly classified without ITSS scoring and thereby reducing the MRI sequences needed for evaluation, scanning time and postprocessing effort. Furthermore, as treatment options differ significantly between lymphoma and glioblastoma, the correct radiologic classification is of great clinical relevance. In this context, our approach allows fast and reliable tumor differentiation and patient treatment can be started as soon as possible to maximize optimal tumor control and thus patient outcome—especially in aggressive glioblastoma [17,18,19].

One major drawback of our study is the relative low number of 74 patients. Another possible limitation is the selective, manual ROI placement in the solid, contrast-enhanced hot-spot tumor area: as some lesions showed several spots with increased CBV, selective ADC assessment was performed in the area with the highest mean CBV value.

In summary, our multiparametric MRI model integrating selective ADC measurement in combination with DSC perfusion imaging enables a reliable and fast radiological differentiation of glioblastoma and primary central nervous system lymphoma in the clinical routine.

## Figures and Tables

**Table 1:** Selected MRI sequence parameters.

	Contrast-Enhanced T1 MP-RAGE	DWI	DSC Perfusion with Leakage Correction
TR (ms)	2200	7600	2010
TE (ms)	2.67	86	30
Flip angle (°)	8	90	90
FOV (mm^2^)	250	230	230
matrix (pixel)	256 × 256	324 × 372	128 × 128
voxel size (mm)	1.0 × 1.0 × 1.0	1.2 × 1.2 × 5	1.8 × 1.8 × 3
acquisition time (min)	4:59	1:25	1:48

TR = repetition time; TE = echo time; FOV = field of view; MP-RAGE = magnetization prepared-rapid gradient echo; DWI = diffusion-weighted imaging; DSC = dynamic susceptibility contrast.

**Table 2:** Classification of GBM and PCNSL using single- and multiparameter models.

	Cut-off	Correctly Classified (%)	Identified GBM Patients	Identified PCNSL Patients	AUC
CBV_min_	4.5	69	35 of 37	16 of 37	0.703
CBV_max_	473	78	29 of 37	29 of 37	0.857
CBV_mean_	129.5	76	27 of 37	29 of 37	0.804
ratio_CBV_	2.2	86	31 of 37	33 of 37	0.930
ADC_sel min_	982	68	13 of 37	31 of 37	0.573
ADC_sel mean_	1066	72	23 of 37	30 of 37	0.762
ADC_sel max_	1314	80	26 of 37	33 of 37	0.847
ADC_unsel min_	642	68	13 of 37	31 of 37	0.516
ADC_unsel mean_	1220	70	19 of 37	33 of 37	0.750
ADC_unsel max_	1973	70	28 of 37	24 of 37	0.722
Multiparameter model	-	100	37 of 37	37 of 37	1
